# Application of the augmented reality tool VSI holomedicine for improved patient education before sinus surgery – a prospective randomised pilot study

**DOI:** 10.1038/s41598-025-21449-w

**Published:** 2026-01-16

**Authors:** Henning Schewe, Hans-Jürgen von Lücken, Patrick M. House, Arne Böttcher, Adrian Münscher

**Affiliations:** 1 Department of Otorhinolaryngology, Head and Neck Surgery, Katholisches Marienkrankenhaus gGmbH, Alfredstrasse 9, Hamburg, 22087 Germany; 2 Specialists Practice for Epileptology, Epileptologicum Hamburg, Leinpfad 75, Hamburg, 22299 Germany; 3 Department of Otorhinolaryngology, University Medical Center Hamburg-Eppendorf, Martinistrasse 52, Hamburg, 20246 Germany

**Keywords:** Augmented reality, Patient education, Functional endoscopic sinus surgery, Hologram glasses, Clinical trials, Randomized controlled trials, Three-dimensional imaging, Respiratory tract diseases, Anatomy, Diseases, Medical research, Medical imaging, Health services, Health care, Patient education

## Abstract

The use of augmented reality (AR) glasses in scientific research but also in medical training and perioperative and intraoperative phases is steadily increasing. To date, there are few high-quality studies available on their use as an educational method prior to surgical interventions. Nonetheless the available data suggests a high potential as a tool in preoperative settings. This study therefore investigated whether the use of the AR tool VSI HoloMedicine as an application on the HoloLens 2 by Microsoft before ESS is superior to the conventional form of patient education using CT images on a PC screen. Over a period of 6 months 20 patients with indications for ESS were included in this prospective randomised controlled clinical study. These were randomized into two subgroups. In both groups, information was provided both using the conventional method and VSI HoloMedicine. The subgroups differed regarding the order in which the two forms of education took place. Following each educational session patients completed a questionnaire. The visualization ability was significantly improved using the new educational method. In direct comparison of the educational methods, a clear majority of patients opted for VSI HoloMedicine as more helpful for the decision to undergo surgery and assessed it as the educational method of the future. VSI HoloMedicine is a promising tool for preoperative education prior to paranasal sinus surgery to improve the patient’s understanding and visual imagination of paranasal sinus surgery. As a result, patient benefit can be increased using the new form patient education.

**Supplementary Information:**

The online version contains supplementary material available at 10.1038/s41598-025-21449-w.

## Introduction

Patient-centred medicine envisages a comprehensively informed patient who comes to a decision on their own health issues together with their doctor^1^. Preoperative information is an important part of fulfilling this ideal. Adequate patient education can improve patient satisfaction, treatment adherence, and treatment success^2^, as well as reduce treatment costs and treatment duration^2,3^. Previous studies have shown that patients only remember a small proportion of the information given to them^4–6^. Reasons for this include low comprehensibility of the information provided and high levels of tension, anxiety and worry^4^. Typically, the perspectives and evaluations of patients and doctors on the educational discussions that have taken place differ greatly^7^. An improvement in the patients’ understanding and level of information can be achieved by supporting the spoken information with visual material^4^. The use of 3D models has proven to be beneficial for this purpose and is associated with a significant improvement in understanding and increasing patient satisfaction^8–10^. Augmented reality (AR) is a form of 3D technology that has recently became accessible to the general public. Here, a computer-generated virtual 3D image is projected onto the user’s view of the real world, thus providing additional information. In a review by Urlings et al. the current state of research regarding the role and effectiveness of AR in patient education was evaluated^11^. They came to the conclusion that even though the research on AR to date is quite limited, it has promising potential for patient education^11^. However, previous literature still contains studies with heterogeneous applications and study populations. Consequently, more high-quality studies are required^11^. The current study addresses this demand and specifically examines patient education before sinus surgery using the AR application VSI HoloMedicine of the company apoQlar GmbH, Hamburg, Germany with the question of patient benefit, reduction of fears and anxieties, the actual level of information and the tendency regarding the decision for medically indicated surgery. This study examines whether the new form of patient education is superior to the standard method of patient education using 2D computed tomography (CT) images on a personal computer (PC) screen.

## Methods

### Design of the study

This randomized prospective clinical trial included 20 patients with indications for endoscopic sinus surgery (ESS). The information collected includes the patients‘ professional status and highest level of education. The number of cases and the distribution within the groups in the current study was confirmed by the Institute for Medical Biometry and Epidemiology of the University Medical Center Hamburg-Eppendorf. Blinding of the examiner or patient was not possible due to the nature of the study design.

The patients were recruited from a collective that presented themselves for surgery planning to the day clinic of the Department for Otorhinolaryngology, Head and Neck Surgery of the Marienkrankenhaus Hamburg in the period from May to October 2023. Included were all patients aged 18 or older of any gender and ethnicity with medical indication for ESS as an initial nasal procedure. Exclusion criteria were unilateral or bilateral anopsia, proven cognitive disability, previous sinus or nasal surgery, and rejection of participation in the study. The study was approved by the ethics committee of the Hamburg Medical Association (processing number: 2022-100990-BO-ff) and is registered with the German Clinical Trial Register (ID: DRKS00035992, date of registration: 01/29/2025). It conforms to the ethical guidelines of the Declaration of Helsinki and Good Clinical Practise. Written informed consent was obtained from all included patients.

The patients were randomized into two groups. The assignment to the groups was conducted via a randomization list (simple randomization) provided by the Institute for Medical Biometry and Epidemiology of the University Medical Centre Hamburg-Eppendorf, Hamburg, Germany. Only a third person who was not involved in the study had access. For both groups, preoperative patient education was provided using the conventional method via 2D images on a PC screen as well as using VSI HoloMedicine. One group received their preoperative education via VSI HoloMedicine first and then filled out the first questionnaire [Appx. 1]. This questionnaire included 10 questions to evaluate the educational method regarding patient satisfaction, reduction of fears and anxieties, and comprehension of the information provided. Additionally, it contained 3 questions evaluating objectively quantifiable knowledge [Appx. 1]. Afterwards, they received preoperative education via 2D images on a PC screen before filling out the final questionnaire to compare both educational methods. This second questionnaire included 2 questions for direct comparison of both educational methods as well as 4 questions in relation to socio-demographic data [Appx. 2]. The first 10 questions for evaluating the educational methods as well as the comparative questions were taken from a similar study done on patient education before epilepsy surgery^12^. The procedure with the second group differed only in terms of the order in which the educational methods took place: Patients first received their preoperative education via 2D images on a PC screen, then filled out the first questionnaire. Afterwards, they were educated via VSI HoloMedicine. For better understanding of the methodology, see the flowchart depicting the procedure [appx. 3].

The CT scans used for both forms of education were conventional low-dose native CT scans of the paranasal sinuses (model used: SOMATOM X.ceed by Siemens, layer thickness: 1 mm, scan range: maxillary dental arch up to and including the frontal sinus, incorporating the nasal tip and the auricle (entire ear)). The educational sessions using both methods took 30 min on average and were performed by the same examiner.

### VSI HoloMedicine

**Fig. 1:**
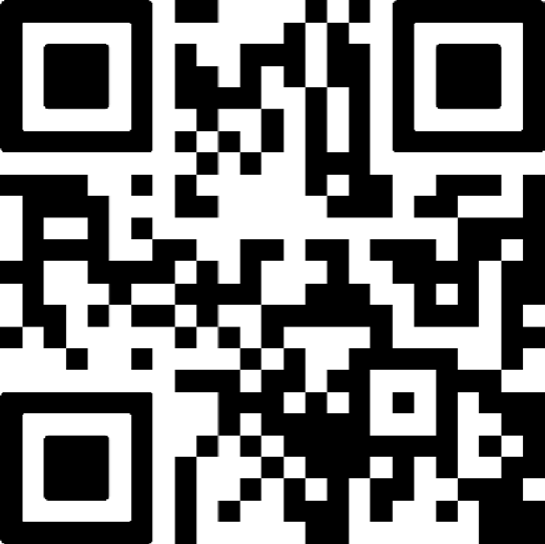
Link to a promotional video for VSI HoloMedicine by apoQlar.

**Fig. 2:**
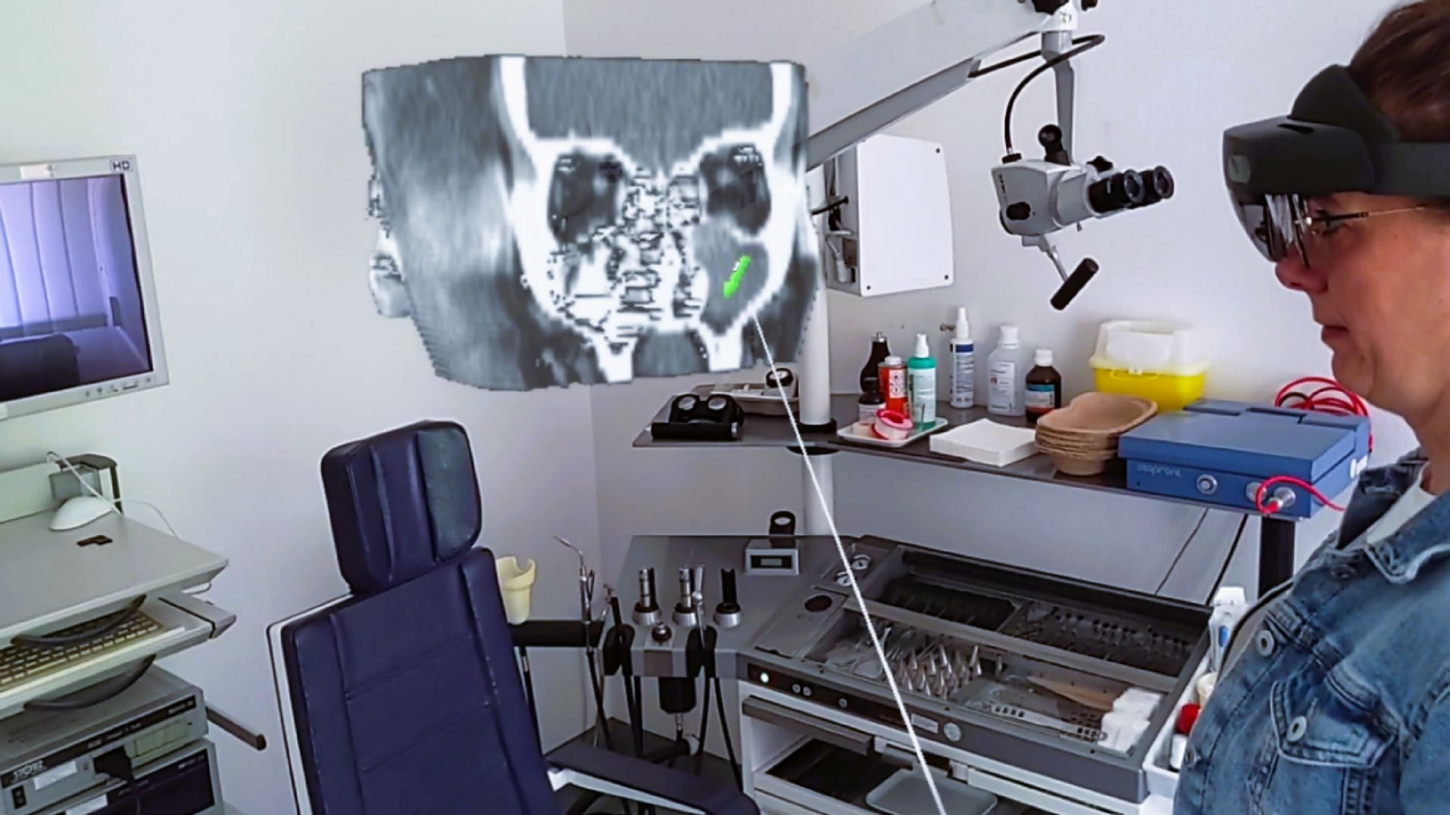
Real educational situation from the perspective of the educator. Both the educator and the patient look at the patient’s own CT scan of the skull as a 3D hologram. Written informed consent was obtained by the participant to publish the image.

**Fig. 3:**
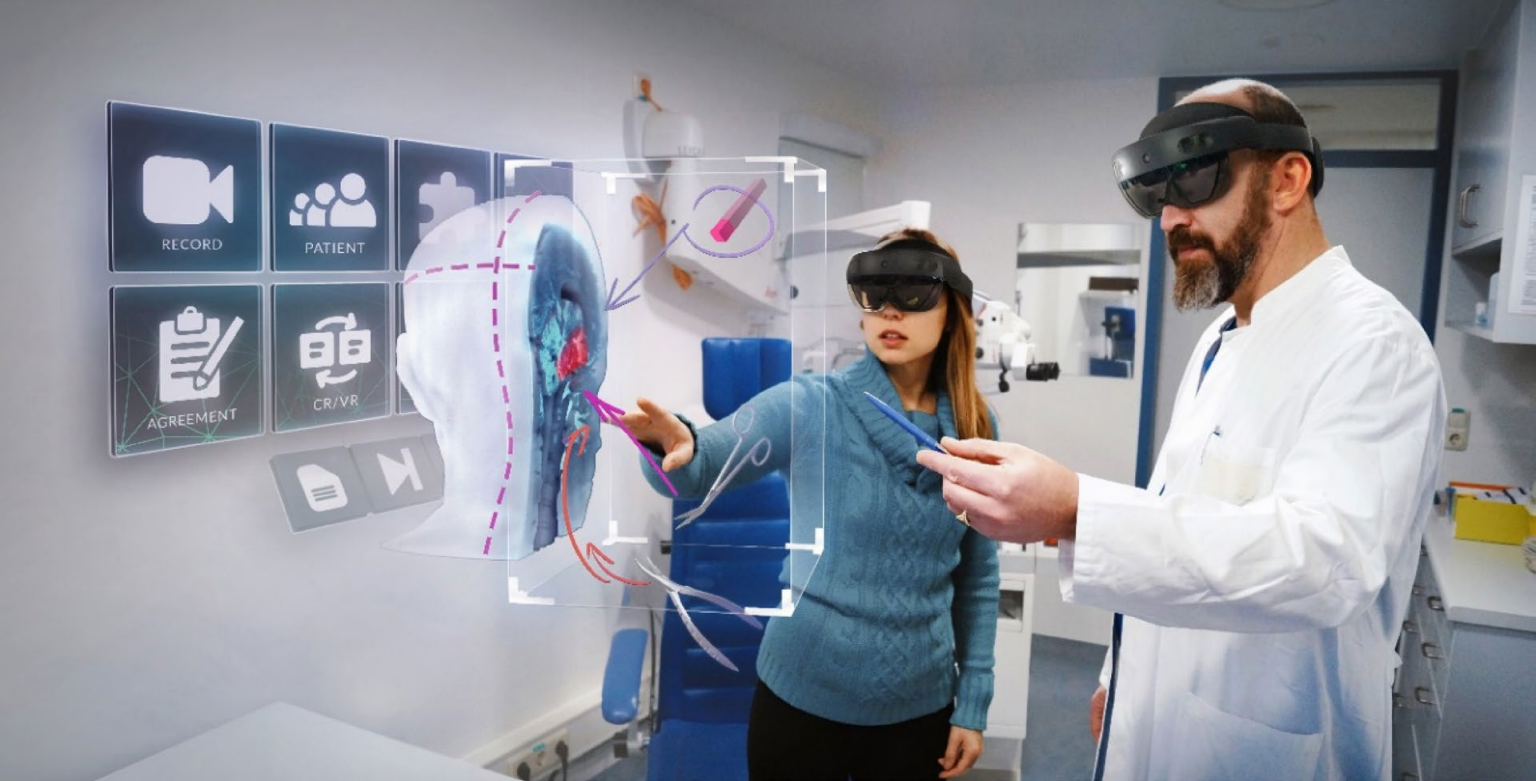
Re-enacted educational situation with schematic representation of a 3D hologram.

VSI HoloMedicine (apoQlar GmbH, Hamburg, Germany, www.apoqlar.com) is a software application used on the AR glasses HoloLens 2 (Microsoft Corporation, Redmond, WA, USA, www.microsoft.com). VSI HoloMedicine enables looking at MRIs or CT scans through a HoloLens 2 from any adjustable perspective in 3D. 3-dimensionality is beneficial for surgeons to plan operations or to explain surgical details to patients in a more visually imaginable way. For a better understanding of VSI HoloMedicine see a promotional video of apoQlar via QR Code [Fig. 1]. In the case of this study, briefly before the educational session, the images of the patient’s own CT scan of the paranasal sinuses were uploaded to VSI HoloMedicine to be used instantly. During the session, both the doctor and the patient wore HoloLenses 2 and, using the “Shared Experience” feature, worked together on the same shared hologram. The 3D object could be enlarged and reduced in size as well as moved through the room by using hand gestures. The doctor was able to inform the patient by using a 3D representation of the patient’s own head and, in particular, the patient’s paranasal sinuses in relation to the upcoming operation, including all anatomical and topographic conditions. Several slicing and marking tools, such as arrows and circles, were employed to point out anatomical or pathological structures [Figs. 2 and 3]. In the context of this study, pointing tools (e.g., slicer and arrows to highlight relevant structures) and pathology-specific imaging planes were used in a standardized way to explain anatomy, pathology, and the surgical procedure to the patients. However, a visualization of the surgical intervention itself - for example, including surgical instruments or depictions of individual operative steps - was not provided.

### 2D CT images on a PC screen

**Fig. 4:**
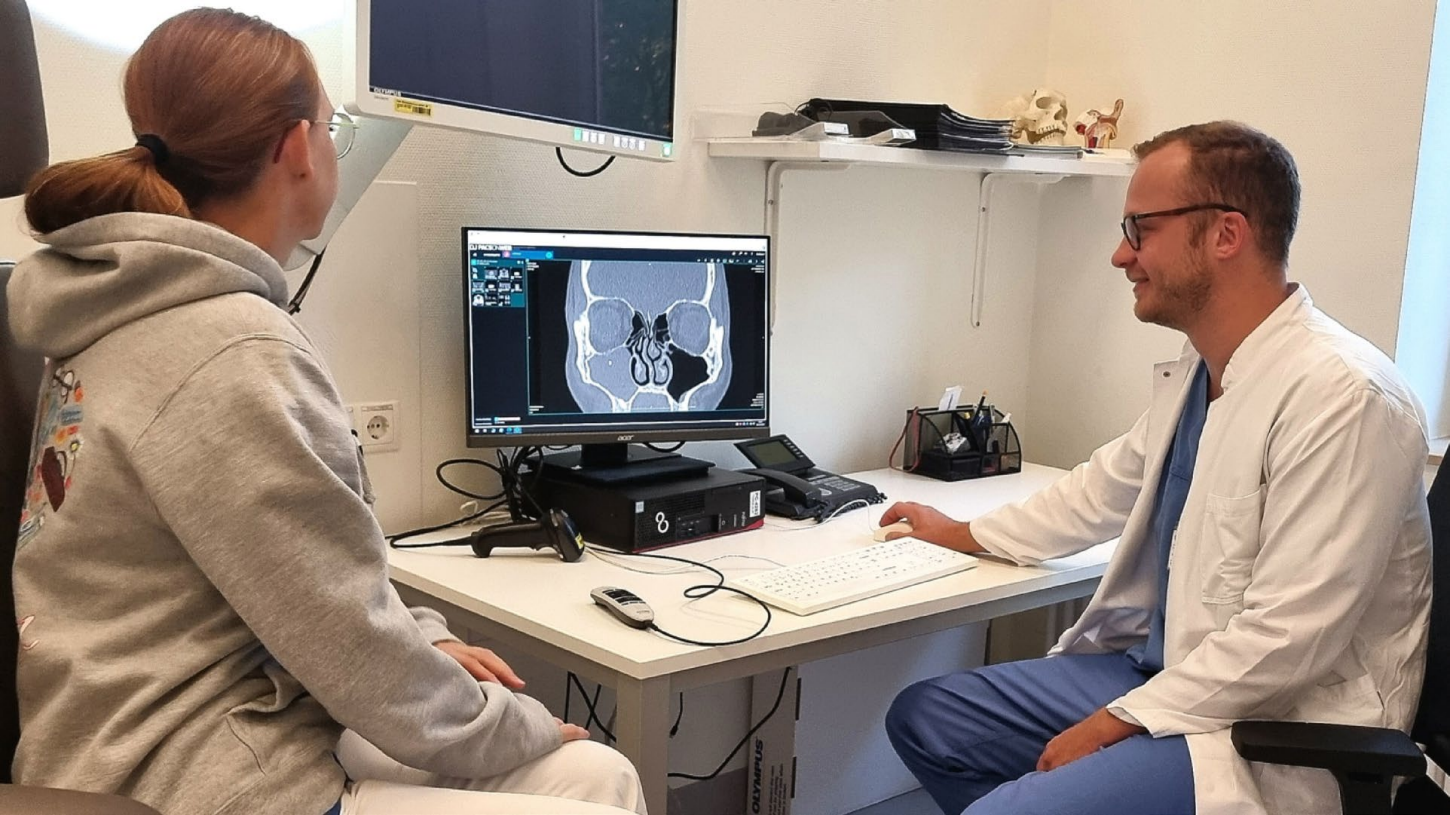
Real educational situation using 2D CT images on a PC screen. Written informed consent was obtained by the participant to publish the image.

Preoperative education via the patient’s own CT images depicted on a PC Screen is a conventional and widely spread method in everyday clinical practice. In this study a B246HL 24” LCD Screen (Acer, Taipeh, Taiwan, www.acer.com) was used to present the 2D CT images. Different sectional planes were demonstrated, and the cursor was used to point out anatomical and pathological structures [Fig. 4]. The operation and the associated risks as well as the potential benefits could consequently be explained in an individualized way.

### Clinical endpoints and statistical analysis

The patient benefit was the primary endpoint of the study. This was measured by questions 1–5, 7 and 9 of the first questionnaire, as well as two questions for direct comparison of the educational methods in the final questionnaire. The secondary endpoints were the reduction of fears and anxieties (questions 6 and 8, 1st questionnaire), the actual level of information after each educational method (questions 11, 12 and 13, 1st questionnaire), and the tendency regarding the decision to have a medically indicated operation (question 10, 1st questionnaire; question 1, 2nd questionnaire).

The collected data was analysed using SPSS (IBM SPSS Statistics 28.0.0., IBM, www.IBM.com, Armonk, USA). The D’Agostino-Pearson test was used to statistically assess the normal distribution. Mean values and standard deviations were calculated. These are given here as mean ± standard deviation for e.g. age. The significance level was calculated using the p-value (*p* ≤ 0.05, highly significant if *p* ≤ 0.01). An analysis of the variables could not prove normal distribution. Therefore, the Mann-Whitney U test was used to evaluate questions 1–10. The 3 subsequent questions regarding the quantifiable knowledge were summarized and classified as “applicable” or “inapplicable” under the variable “all questions answered correctly”. The evaluation was carried out using Fisher’s exact test. Socio-demographic data and comparative questions were analysed descriptively.

## Results

The patient collective consisted of 20 patients aged 46,8 y ± SD 16,45 y (range: 22–80 y). Of these, 8 patients were between 22 and 39 years old, 6 patients were between 40 and 59 years old and 6 patients were between 60 and 80 years old. The group that was primarily educated via the standard method consisted of 12 patients aged 43,8 y ± SD 18,4 y (range: 22–80 y, median: 39,5 y, 95% CI: 32.2–55.5 y). The group that was primarily educated via AR consisted of 8 patients aged 47,5 y ± SD 14.3 y (range: 30–65 y, median: 49.5 y, 95% CI: 35.5–59.5 y). According to the patients’ information on their highest educational qualification and occupationall levels of the International Standard Classification of Occupation (ISCO) were represented apart from managers and armed forces occupations. The group educated via AR consisted of the ISCO major groups 2, 3, 5, and 7 (professionals, technicians and associate professionals, service and sales workers, craft and related trades workers). The group educated via the standard method consisted of ISCO major groups 2, 3, 4, 5, 6, 8 and 9 (professionals, technicians and associate professionals, clerical support workers, service and sales workers, skilled agricultural, forestry and fishery workers, plant and machine operators, and assemblers, elementary occupations). In both groups, educational qualifications, ranging from the German lower secondary school leaving certificate (*Hauptschulabschluss*) to a master’s degree, were recorded. One observation made by the examiner was that despite a heterogeneous patient population, all patients accepted the new educational method using VSI HoloMedicine on the HoloLens2 and were able to handle it intuitively after 2–3 min of short introduction.

In the evaluation of the answers to questions 1–3 and 5–10 [Appx. 1], no statistically significant difference was found between the patient groups (q.1: *p* = 1,0; q.2: *p* = 0,41; q.3: *p* = 1,0; q.5: *p* = 0,27; q.6: *p* = 0,33; q.7: *p* = 0,17; q.8: *p* = 0,38; q.9: *p* = 0,63, q.10: *p* = 0,86). It should be noted that only 3.5% of all patients in both groups chose the “third best” answer [Table 1]. No patients elected the “fourth best”, i.e. the least best answer. Therefore, most questions (96.5%), were answered with either the “best” or “second-best” answer option. For question 4 (“Were you able to visualize the operation well with the help of the demonstration?“), a statistically significant difference was found between the two groups using the Mann-Whitney U-test (*p* = 0,046, U = 26,0, Z = −1,99). Here, the group of patients who were educated using AR glasses reported a better visualization of the operation. However, this statistical significance only exists under the assumption of a sufficient population size (asymptotic significance). Using exact significance, the result is just below the required level of statistical significance (*p* = 0,098).

When looking at the 3 questions to evaluate quantifiable knowledge, the study found that only 9 out of 12 patients (i.e. 75%) who had received the standard education method answered all the questions correctly. In the group that had received the novel education method, 8 out of 8 patients (i.e. 100%) answered all questions correctly [Table 2]. However, this difference could not be proven to be statistically significant.

At the end of the session, the study participants were asked two questions for direct comparison of the two educational methods. In response to the question “Which educational method helped you more in your decision for or against an operation?“, 90% of patients (18 out of 20) opted for the educational method using VSI HoloMedicine [Table 3]. When asked “Which information method should be used as the standard method in the future?“, 95% (19 out of 20) of patients also chose the new educational method using AR glasses [Table 3].

**Table 1:** Distribution of answers to questions 1–10 of the 1 st questionnaire (preoperative education via VSI holomedicine vs. 2D images on a PC screen), question 4 marked with * shows statistically difference in favour of the VSI holomedicine group (asymptotic significance: 0,46).

Question	Share in % - Best answer		Share in % - 2nd best answer		Share in % - 3rd best answer		Share in % - Least best answer	
	VSI HoloMedicine	PC screen	VSI HoloMedicine	PC screen	VSI HoloMedicine	PC screen	VSI HoloMedicine	PC screen
Did you have the opportunity to ask questions during the patient education?	100	100	0	0	0	0	0	0
Were your questions about the operation answered?	100	91,6	0	8,3	0	0	0	0
Was the explanation easy for you to understand?	100	100	0	0	0	0	0	0
Were you able to visualize the operation well with the help of the demonstration? *	87,5	50	12,5	50	0	0	0	0
Do you feel that this form of patient education method is state of the art?	62,5	41,6	37,5	41,6	0	16,6	0	0
Has this educational method reduced your fears/anxieties about surgery?	12,5	41,6	87,5	50	0	8,3	0	0
How well informed do you currently feel about the operation?	87,5	58,3	12,5	41,6	0	0	0	0
How big do you think your concerns/fears/anxieties about the operation are at the moment?	0	8,3	100	66,7	0	25	0	0
How well do you currently feel supported in the decision regarding the operation?	75	75	25	25	0	0	0	0
How confident are you at the moment in your decision for or against an operation?	50	58,3	50	33,3	0	8,3	0	0

**Table 2:** Distribution of answers to knowledge questions of 1 st questionnaire.

Question	Share in % - correct		Share in % - incorrect	
	VSI HoloMedicine	PC screen	VSI HoloMedicine	PC screen
Where are the nasal conchae located?	100	75	0	25
Which structure is at risk of being injured during surgery on the paranasal sinuses?	100	100	0	0
Which structure may also need to be operated on in order to reach the paranasal sinuses with the surgical instruments?	100	91,6	0	8,3

**Table 3:** Distribution of answers to comparative questions in final questionnaire.

Question	Share in %	
	VSI HoloMedicine	2D images on a PC screen
Which educational method helped you more in your decision for or against an operation?	90	10
Which information method should be used as the standard method in the future?	95	5

## Discussion

This study indicates that the AR application VSI HoloMedicine can make a valuable contribution toward achieving the ideal of the comprehensively informed patient. Other studies have already shown that patient education can be improved with the help of complementary visual materials^4^. Accordingly, AR as a tool exhibits great educational potential in medical consultations with a positive effect on knowledge retention and patient satisfaction, as shown in meta-analyses^11^. The present study shows that VSI HoloMedicine can enhance patients’ visual imagination of anatomy and surgical procedures. The AR application is superior to the standard procedure, which relies on a 2D PC screen as visual aid. This is in line with the results of the study “Use of the mixed reality tool ‘VSI Patient Education’ for more comprehensible and imaginable patient education before epilepsy surgery and stereotactic implantation of DBS or stereo-EEG electrodes” by House et al.^12^. Here, the predecessor to VSI HoloMedicine was compared to patient education supported by the use of a 3D anatomical model of the skull and brain^12^. They found VSI HoloMedicine to be the more effective educational tool as well^12^. The results of our study are also consistent with those of the aforementioned colleagues regarding the direct comparison of educational methods. In general, the main purpose of preoperative patient education is to support patients in their decision-making^1^. In the questionnaire for the present study, the significant majority of patients chose the new AR-based method as the answer to “Which educational method helped you more in your decision for or against an operation?”, and “Which educational method should be used as the standard method in the future?”. The fact that an overwhelming majority of patients (90%) rated VSI HoloMedicine as more helpful in this decision-making process demonstrates great potential for patient care and patient satisfaction. This promise for patient benefit is underlined by the fact that almost all patients (95%) chose that the AR tool should be the new standard method in the future. All participating patients opted for the medically indicated operation. This result could be indicative of the fact that a more comprehensively educated patient (via AR) will ultimately decide in favour of the medically indicated surgery.

Nevertheless, one discrepancy is apparent here. Except for question 4 (“Were you able to visualize the operation well with the help of the demonstration?“), no statistically significant difference for all questions from the 1st questionnaire could be found. This entails that on the one hand, there is limited statistically significant difference when comparing the two groups and how they evaluated the respective educational method. On the other hand, the clear majority opted for the new educational method using AR glasses in direct comparison after having undergone both forms of patient education. A reason for this discrepancy could be that patients might generally appreciate a high-quality patient education. It is well understood that other factors - beyond the choice of educational tools - significantly impact the quality of patient education. Clinical experience demonstrates that the physician’s ability to dedicate sufficient time to the patient, to respond comprehensively to all questions, and to address patients’ concerns and anxieties with empathy plays a pivotal role^2^. In this present study, this potential bias was addressed by ensuring that all consultations were conducted by the same physician and that the educational content was the same across both groups, irrespective of the tool employed. Nevertheless, these biases may still have affected the way participants responded to the questionnaires. This effect may be more pronounced for certain issues addressed in the questionnaire, since some questions are more generally aimed at the quality of patient education than others. For example, “Did you have the opportunity to ask questions during the patient education?” could be influenced more by the aforementioned factors of patient-doctor communication than others such as “Were you able to visualize the operation well with the help of the demonstration?“. The questionnaire items were phrased as such to ensure comparability with the previously cited study by House et al.^12^. A generally high quality of patient education in this study may be reflected in the fact that 96.5% of patients chose the “best” or “second best” answer to all questions. This may reveal another underlying general problem when it comes to evaluating a new technique for preoperative patient education that is unknown to the general population. The patients in the group who received the conventional form of education using 2D CT images on a PC screen evaluated the educational method based on their previous experience. It is highly likely that they have not yet had any contact with AR - certainly not in a hospital setting. Accordingly, they are also unfamiliar with the new possibilities of visualization using 3D holograms and might not notice possible deficits in the usage of 2D images, leading to a generally positive evaluation of the conventional form of patient education. However, this does not indicate that patients cannot benefit from pre-surgery education via AR. They clearly prefer the new method once they are aware of both methods – as reflected in the comparative questions.

Regarding fears and anxieties related to the upcoming operation, the question arises whether an enhanced ability to visualize the procedure might actually increase such emotions. However, the collected data from the questionnaires showed no significant difference between the patient groups in that matter. Nonetheless, from discussions with patients during the study, the medical provider deduced that improved visualization might lead to more concerns. For example, the anatomical proximity of the surgical site and instruments to sensitive structures to the surgical site, such as the eye, becomes tangible through better visualization using 3D holograms. However, concealing such anatomical properties from the patient goes against the goal of achieving informed consent. Rather, the surgeon has the task of realistically presenting the surgical risk to the patient and thus supporting them in making an informed decision^1^.

There are limitations to the current study. The above-mentioned statistically significant difference regarding the enhancement of visual imagination (question 4, 1st questionnaire) only exists under the assumption of a sufficient population size. The population of 20 patients is possibly borderline in this context. The sample size chosen for this study was based on several considerations: First, in consultation with the Institute for Medical Biometry and Epidemiology of the University Medical Center Hamburg-Eppendorf, a sample size comparable to that used in the aforementioned study by House et al.^12^ was selected, as statistically significant results were obtained in that study. Furthermore, the present study was conceived as a pilot study aimed at testing a novel technology in a clinical setting. This study may serve as a foundation for future, more expansive research. Future studies with larger sample sizes might be useful to allow for a more detailed and differentiated evaluation of the presented approach. Ultimately, it cannot be assumed with certainty that a larger sample size would have yielded more significant results within the present study design, as the aforementioned potential bias associated with the novelty of the technique may still have influenced the findings.

The absolute difference in answering the questions of knowledge (75% vs. 100% answered all questions correctly) may indicate an improved level of knowledge on the part of the group educated using VSI HoloMedicine. However, this difference is not statistically significant and may be subject to bias (level of education, IQ, prior knowledge, etc.). Although the data analysis in this study revealed a wide variation of educational levels, no statistical conclusions can currently be drawn from this due to the sample size, nor can a potential bias be ruled out. To address this issue in more detail, further studies are needed.

In this study, the AR tool VSI HoloMedicine was used as a potential tool for patient-specific 3D visualization but there are of course various approaches to patient-specific 3D visualization. In addition to AR, such tools as virtual reality (VR), 3D printing, and 3D visualizations on computer screens have also gained traction in the medical field as valuable and valued tools for patient information. To identify the “optimal” method for preoperative patient education, further comparative studies that include different 3D tools are warranted. However, the aim of the present study was to initially assess a specific, novel AR tool in comparison to the standard approach using 2D images on a PC screen. A potential limitation of this study is that patients’ preference for the AR-based educational method may have been influenced by the novelty of the technology itself.

From a statistical perspective, a finer interval scaling of the response options in the first questionnaire might have been advantageous. This could have made differences between the groups clearer. However, it was decided to use the same questionnaire as in the study by House et al. to ensure comparability^12^.

An important aspect in the implementation of AR technology is its applicability to everyday clinical practice. The use of AR tools for patient education should not disrupt clinical workflows and, ideally, should contribute to their improvement over time – however, this technology will at first be unfamiliar to most doctors. Based on this study’s investigator’s own experience with the equipment, following a brief initial phase of technical familiarization, the setup of the educational scenario (including launching the AR glasses, uploading patient-specific CT images, and generating the 3D CT hologram) using VSI HoloMedicine should not pose significant challenges to any doctor who regularly provides patients with information.

Since high-quality patient education can improve treatment adherence and outcomes^2^ and reduce both treatment costs and duration^2,3^, the use of AR tools would most likely have a positive impact on these factors. It appears generally conceivable that AR will play an increasingly important role not only in preoperative settings but also in intraoperative contexts^13,14^. Future studies will be needed to determine in which specific areas of application this technology can provide additional benefit, and where its limitations may become apparent.

## Conclusion

In summary, patients prefer the innovative educational method using VSI HoloMedicine as the standard method of the future and consider it more helpful when deciding on medically indicated surgery. The ability to visualize the operation, the anatomy and the resulting risks appears to be improved using AR glasses. With this tool, an increased patient benefit and satisfaction will likely be achieved. Further, more expansive studies that are dedicated to the use of AR in the context of preoperative patient education are needed. AR in general and VSI HoloMedicine in particular are a promising tool that has the potential to have a decisive impact on everyday clinical practice in the future.

## Supplementary Information

Below is the link to the electronic supplementary material.

Supplementary Material 1

Supplementary Material 2

Supplementary Material 3

Supplementary Material 4

Supplementary Material 5

## Data Availability

The datasets generated during and/or analysed during the study are available from the corresponding author on reasonable request.
