# Supplementary material for: Application of the augmented reality tool VSI holomedicine for improved patient education before sinus surgery – a prospective randomised pilot study
Source: Sci Rep. 2026 Jan 16;16:6371. doi: 10.1038/s41598-025-21449-w (PMC12905279; doi:10.1038/s41598-025-21449-w)
Supplement: Supplementary file 1 — Supplementary Material 1 [file 41598_2025_21449_MOESM1_ESM.docx]

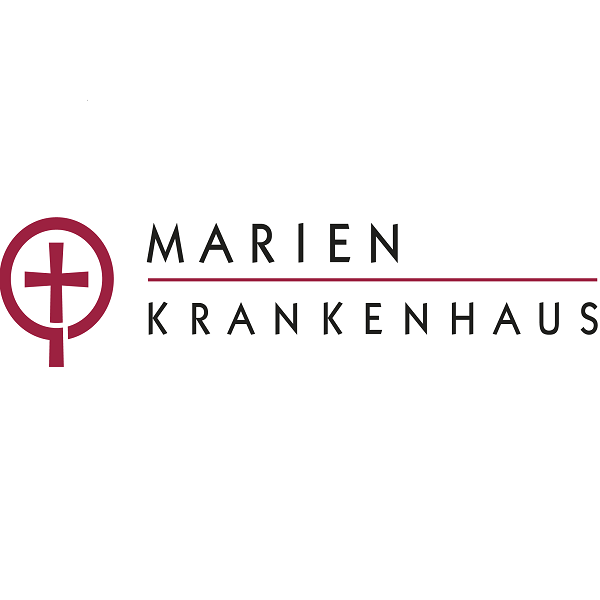


**Study: Application of the augmented reality tool “VSI HoloMedicine®” on the HoloLens 2 for more vivid patient education before endoscopic sinus surgery (ESS)**

**Patient questionnaire**

| You have just been informed about FESS (functional endoscopic sinus surgery) for the treatment of chronic sinusitis. CT images on the PC screen/augmented reality glasses were used for the patient education.  We now ask you to evaluate this method: |
| --- |
| 1. Did you have the opportunity to ask questions during the patient education? |
| □ yes |
| □ mostly |
| □ partly |
| □ no, not at all |
| 1. Were your questions about the operation answered? |
| □ yes, in full |
| □ mostly |
| □ partly |
| □ no, not at all |
| 1. Was the explanation easy for you to understand? |
| □ yes, very comprehensible |
| □ partially comprehensible |
| □ only slightly comprehensible |
| □ incomprehensible |
| 1. Were you able to visualize the operation well with the help of the demonstration? |
| □ yes, very good indeed |
| □ Yes, partly, but not everything |
| □ only little |
| □ no |
| 1. Do you feel that this form of patient education method is state of the art? |
| □ yes, in full |
| □ mostly |
| □ partly |
| □ no, not at all |
| 1. Has this educational method reduced your fears/anxieties about surgery? |
| □ yes, in full |
| □ mostly |
| □ partly |
| □ no, not at all |
| 1. How well informed do you currently feel about the operation? |
| □ not at all |
| □ a little, but not yet enough |
| □ sufficiently |
| □ in full |
| 1. How big do you think your concerns / fears / anxieties about the operation are at the moment? |
| □ no concerns at all |
| □ few concerns |
| □ major concerns |
| □ very big concerns |
| 1. How well do you currently feel supported in the decision regarding the operation? |
| □ not at all |
| □ just a little |
| □ sufficiently |
| □ in full |
| 1. How confident are you at the moment in your decision for or against an operation? |
| □ very uncertain |
| □ uncertain |
| □ certain |
| □ very certain |

**Questions of knowledge**

| 1. Where are the nasal turbinates located? |
| --- |
| □ above the eyes |
| □ in the maxillary sinuses |
| □ in the main nasal cavities |
| □ behind the nasal septum |
| 1. Which structure is at risk of being injured during surgery on the paranasal sinuses? |
| □ the ear |
| □ the eye |
| □ the parotid gland |
| □ the larynx |
| 1. Which structure may also have to be operated on in order to reach the paranasal sinuses with the surgical instruments? |
| □ the teeth |
| □ the nasal septum |
| □ the bridge of the nose |
| □ the tip of the nose |
