# Supplementary material for: Application of the augmented reality tool VSI holomedicine for improved patient education before sinus surgery – a prospective randomised pilot study
Source: Sci Rep. 2026 Jan 16;16:6371. doi: 10.1038/s41598-025-21449-w (PMC12905279; doi:10.1038/s41598-025-21449-w)
Supplement: Supplementary file 2 — Supplementary Material 2 [file 41598_2025_21449_MOESM2_ESM.docx]

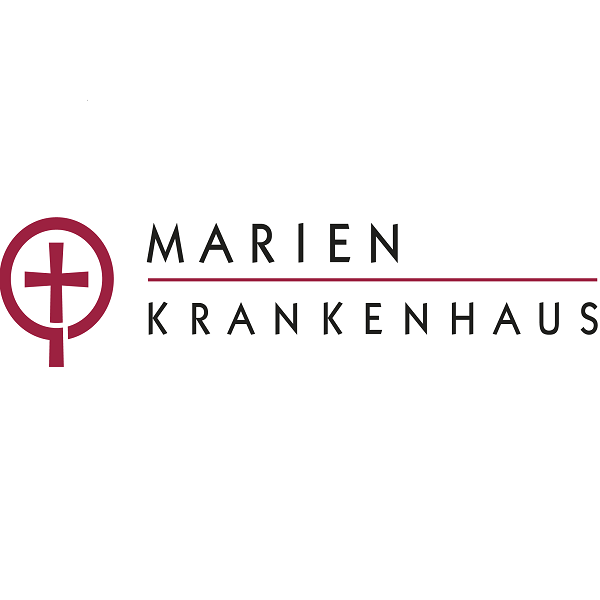


**Study: Application of the augmented reality tool “VSI HoloMedicine®” on the HoloLens 2 for more vivid patient education before endoscopic sinus surgery (ESS)**

| Which educational method helped you more in your decision for or against an operation? | |
| --- | --- |
| □ augmented reality glasses | □ CT images on the PC screen |

**Final questionnaire**

| Which educational method should be used as the standard method in the future? | |
| --- | --- |
| □ augmented reality glasses | □ CT images on the PC screen |

**Age: ­­ ­_______ years**

**Sex:** □ m □ f □ d

**Occupation: __________________________________________________________________________________________**

**Highest educational qualification: __________________________________________________________________________________**
