# Supplementary material for: Application of the augmented reality tool VSI holomedicine for improved patient education before sinus surgery – a prospective randomised pilot study
Source: Sci Rep. 2026 Jan 16;16:6371. doi: 10.1038/s41598-025-21449-w (PMC12905279; doi:10.1038/s41598-025-21449-w)
Supplement: Supplementary file 3 — Supplementary Material 3 [file 41598_2025_21449_MOESM3_ESM.docx]

**Appendix 4**

Flowchart depicting the procedure and methodology

Statistical analysis

2nd questionnaire

Randomization, n=20

patient education via standard method (2D images on PC screen)

Patient education via AR tool VSI HoloMedicine

Patient education via standard method (2D images on PC screen)

1st questionnaire

Intervention group, n = 12

Intervention group, n = 8

Patient education via AR tool VSI HoloMedicine
