# Supplementary figures and images for: Application of the augmented reality tool VSI holomedicine for improved patient education before sinus surgery – a prospective randomised pilot study
Source: Sci Rep. 2026 Jan 16;16:6371. doi: 10.1038/s41598-025-21449-w (PMC12905279; doi:10.1038/s41598-025-21449-w)

**Appendix 4**

Link to promotional Video of apoQlar explaining the use of VSI HoloMedicine


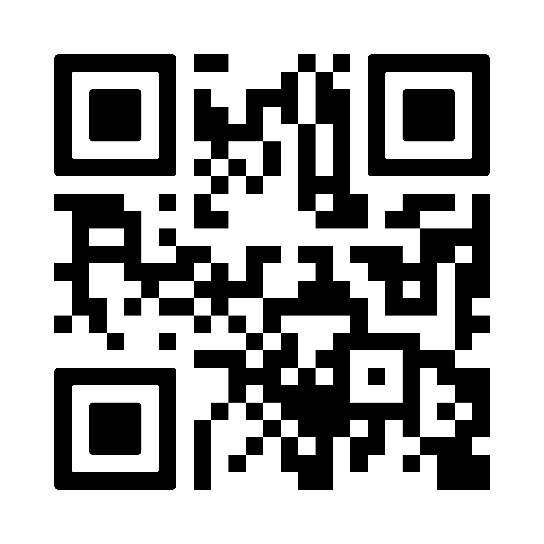


**Fig. 1**

Supplement: Supplementary file 4 — Supplementary Material 4 [file 41598_2025_21449_MOESM4_ESM.docx]
